# Influence of Hesperidin on the Systemic and Intestinal Rat Immune Response

**DOI:** 10.3390/nu9060580

**Published:** 2017-06-06

**Authors:** Mariona Camps-Bossacoma, Àngels Franch, Francisco J. Pérez-Cano, Margarida Castell

**Affiliations:** 1 Section of Physiology, Department of Biochemistry and Physiology, Faculty of Pharmacy and Food Science, University of Barcelona (UB), 08028 Barcelona, Spain; marionacamps@ub.edu (M.C.-B.); angelsfranch@ub.edu (À.F.); franciscoperez@ub.edu (F.J.P.-C.); 2 Nutrition and Food Safety Research Institute (INSA-UB), 08921 Santa Coloma de Gramenet, Spain

**Keywords:** antibody, flavanone, flavonoids, hesperidin, immune system, immunoregulatory, polyphenol

## Abstract

Polyphenols, widely found in edible plants, influence the immune system. Nevertheless, the immunomodulatory properties of hesperidin, the predominant flavanone in oranges, have not been deeply studied. To establish the effect of hesperidin on in vivo immune response, two different conditions of immune system stimulations in Lewis rats were applied. In the first experimental design, rats were intraperitoneally immunized with ovalbumin (OVA) plus *Bordetella pertussis* toxin and alum as the adjuvants, and orally given 100 or 200 mg/kg hesperidin. In the second experimental design, rats were orally sensitized with OVA together with cholera toxin and fed a diet containing 0.5% hesperidin. In the first approach, hesperidin administration changed mesenteric lymph node lymphocyte (MLNL) composition, increasing the TCRαβ+ cell percentage and decreasing that of B lymphocytes. Furthermore, hesperidin enhanced the interferon (IFN)-γ production in stimulated MLNL. In the second approach, hesperidin intake modified the lymphocyte composition in the intestinal epithelium (TCRγδ+ cells) and the lamina propria (TCRγδ+, CD45RA+, natural killer, natural killer T, TCRαβ+CD4+, and TCRαβ+CD8+ cells). Nevertheless, hesperidin did not modify the level of serum anti-OVA antibodies in either study. In conclusion, hesperidin does possess immunoregulatory properties in the intestinal immune response, but this effect is not able to influence the synthesis of specific antibodies.

## 1. Introduction

Polyphenols are secondary metabolites of plants that are widely distributed in fruits (e.g., apple, grape, pear, cherry, berries), vegetables, nuts, flowers, cereals, legumes, chocolate, and beverages (tea, coffee, and wine) [1]. Polyphenols, named thus for the presence of various phenolic groups [2], are mainly classified according to their chemical structure into flavonoids (isoflavones, neoflavonoids, chalcones, flavones, flavonols, flavonones, flavononols, flavanols, proanthocyanidins, and anthocyanidins) or non-flavonoids (phenolic acids or phenolic amides) [3]. 

In the last 20 years, polyphenols have gained attention mainly due to their antioxidant properties [3,4], and a large number of beneficial effects have been reported such as on degenerative disease, cardiovascular disease, cancer, osteoporosis; and their influence on the immune system has also been shown [2,5,6]. Focusing on polyphenol immunomodulatory properties, a number of in vitro, in vivo, and clinical studies have confirmed the influence of various flavonoids on the innate and acquired immune response by attenuating immune function, thus showing their beneficial role in immune hypersensitivity [7]. Accordingly, flavonoid administration has been demonstrated to be useful in the prevention of allergic asthma and rhinitis [8]. 

Hesperidin (5,7,3-trihydroxy-4-methoxyflavanone-7-rhamnoglucoside) is a flavonoid belonging to the flavanone class [9], its aglycone form being hesperetin [10]. Hesperidin is mainly found in the fruits of the genus *Citrus* [1], particularly in the epicarp, mesocarp, endocarp, and juice of citrus fruits [11] and it is the predominant flavanone found in oranges [12,13]. The majority of the flavonoids found in citrus fruits are glycosides and just a little quantity of hesperitin is present [12].

To date, several pharmacological effects of hesperidin have been reported. It prevents hypercholesterolaemia and fatty liver [14], osteoporosis [15], hypertension, and cerebral thrombosis, among others [16]. In terms of its effects on the immune system, the role of hesperidin has been described in reducing Th2 cytokines in mouse models of asthma [9,17] and in stimulated macrophages [18]. Nevertheless, there are no in-depth studies concerning hesperidin’s effect on immune tissues, including the intestinal lymphoid tissue, and on specific antibody synthesis. In this line, the study of such effects on animal models is of interest because it allows the arrival of hesperidin or its metabolites to the lymphoid tissues, and its analysis will contribute to a better understanding of a flavanone-enriched diet on human health. For this reason, the aim of the current study was to spotlight the effects of hesperidin on Th2 antibody production and on lymphoid tissues, focusing on the gut-associated lymphoid tissue (GALT), which is the first line of defence encountered by the hesperidin present in food. We have investigated this action under two different conditions triggering Th2 immune responses and using three different hesperidin dosages. 

## 2. Materials and Methods 

### 2.1. Chemicals 

Hesperidin was provided by Ferrer HealthTech (Murcia, Spain), with a purity of 95.5% (High Performance Liquid Chromatography) containing 2% isonaringine, 1.5% didimine, and other impurities. 

Carboxymethylcellulose (CMC), cholera toxin (CT), fetal bovine serum (FBS), L-glutamine, ovalbumin (OVA, grade V), penicillin-streptomycin, toxin from *Bordetella pertussis* (Bpt), and RPMI 1640 medium were provided by Sigma-Aldrich (Madrid, Spain). Imject™ alum adjuvant was obtained from Thermo Fisher Scientific (Barcelona, Spain). Biotin-conjugated anti-rat immunoglobulin (Ig)A, IgG1, IgG2a, IgG2b, and IgG2c monoclonal antibodies, anti-rat IgE monoclonal antibody, and anti-rat fluorochrome-conjugated monoclonal antibodies (detailed later) were purchased from BD Biosciences (Madrid, Spain), Biolegend (San Diego, CA, USA), or Novus Biologicals (Littleton, CO, USA). Peroxidase conjugated and unconjugated goat anti-rat IgA antibody and IgA standard were provided by Bethyl Laboratories (Montgomery, TX, USA). Peroxidase-conjugated anti-rat Ig was from DakoCytomation (Glostrup, Denmark). 2-β-mercaptoethanol was from Merck (Darmstadt, Germany). Ketamine was provided by Merial Laboratories S.A. (Barcelona, Spain) and xylazine by Bayer A.G. (Leverkusen, Germany).

### 2.2. Animals and Experimental Designs

Three-week-old Lewis rats (Janvier Labs, Saint Berthevin CEDEX, France) were maintained at the animal facility of the Faculty of Pharmacy and Food Science (University of Barcelona) housed in cages (three rats per cage) and kept under controlled conditions of temperature and humidity in a 12 h light-dark cycle. Animal procedures were approved by the Ethical Committee for Animal Experimentation at the University of Barcelona (CEEA/UB ref. 5988) and conducted in compliance with the Guide for the Care and Use of Laboratory Animals. 

**Figure 1:**
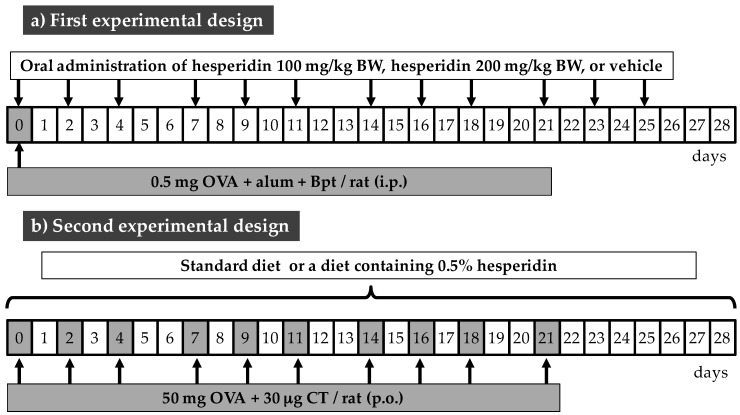
Experimental designs. (**a**) First experimental design: rats were immunized by intraperitoneal (i.p.) route the first day of the study (day 0), and hesperidin was given by oral gavage three times per week (indicated by arrows) for 4 weeks; (**b**) Second experimental design: rats were sensitized by oral route (*per os*, p.o.) three times per week (indicated by arrows), and hesperidin was included in the rat food throughout the 4 weeks. BW, body weight; OVA, ovalbumin; CT, cholera toxin.

The effect of hesperidin on systemic and intestinal immune response was studied in two experimental designs (Figure 1). The first design studied the influence of hesperidin in a systemic immune response that was triggered by an intraperitoneal (i.p.) immunization, as previously described [19]. Briefly, rats received an i.p. injection with 0.5 mg of OVA plus 50 ng of *Bordetella pertussis* toxin (Bpt) in 0.5 mL of alum emulsion (1:3 alum:OVA+Bpt solution). Hesperidin was given by oral gavage three times per week at doses of 100 or 200 mg/kg of rat body weight (BW). Therefore, the first experimental design included three groups: the reference immunized group (OVAip group), the immunized group given 100 mg/kg hesperidin (H100 group), and the immunized group given 200 mg/kg hesperidin (H200 group). Hesperidin was prepared daily in 0.5% CMC as vehicle. The OVAip group received the vehicle. In the second design, the effect of hesperidin on the intestinal immune response was triggered in orally sensitized rats and was included in the rat food. For this, rats were orally sensitized with OVA and CT, as previously described [20], and animals were fed either a standard diet (AIN-93M, Harlan Teklad, Madison, WI, USA) (reference sensitized group: OVAoral group), or a diet containing 0.5% hesperidin (H0.5 group). In both designs, the animals had free access to water and food throughout the study. The consumption of water and food per cage was periodically registered and referred to as water or food consumed per 100 g of BW of the rats included in the cage.

### 2.3. Sample Collection and Processing

At the end of both studies, animals were anaesthetized by subcutaneous route with ketamine-xylazine. Apart from faecal and blood samples, the mesenteric lymph nodes (MLN) and the small intestine were collected. In the second design, the duodenal part of the intestine was discarded and the rest was opened lengthwise in order to separate the Peyer’s patches (PP). From the ileum, intraepithelial lymphocytes (IEL) and lamina propria lymphocytes (LPL) were isolated, as reported previously [21]. From the resting tissue, gut lavage was obtained as previously described [21] and kept at −20 °C for IgA quantification and at −80 °C for cytokine determination.

The lymphocytes from MLN (MLNL) and from PP (PPL) were also isolated, as detailed in prior research [21,22]. Isolated lymphocyte counts and viability were determined by a Countess™ Automated Cell Counter (Invitrogen™, Thermo Fisher Scientific, Barcelona, Spain) in order to proceed with the staining for the flow cytometric analysis or the culture of MLNL. 

Blood samples were centrifuged and serum was kept at −20 °C for antibody determination. From faeces, faecal homogenates supernatants were obtained and kept at −20 °C for intestinal IgA quantification. Briefly, faeces were dried, weighed, diluted with PBS pH 7.2 (20 mg/mL), homogenized with a Polytron (Kinematica, Lucerne, Switzerland), and, finally, the supernatants obtained after centrifugation were kept at −20 °C for intestinal IgA quantification. 

### 2.4. Lymphocyte Phenotypic Analysis

MLNL, PPL, IEL, and LPL were stained with fluorescent-labelled antibodies, as previously described [22]. The following fluorochrome-conjugated antibodies were used: FITC-TCRαβ, FITC-CD8β, FITC-CD25, FITC-TLR4, FITC-CD103, PE-NKR-P1A, PE-TCRγδ, PE-TLR4, PerCP-CD8α, APC-CD4, and APC-Cy8-CD45RA. Data were acquired by Gallios Cytometer (Beckman Coulter, Miami, FL, USA) in the Scientific and Technological Centers of the University of Barcelona (CCiTUB) and the analysis was performed with FlowJo v.10 software (Tree Star, Inc., Ashland, OR, USA). Results are expressed as percentages of positive cells in the lymphocyte population selected according to their forward and side scatter characteristics. 

### 2.5. Specific Anti-OVA Antibodies and Intestinal IgA Quantification

The levels of the specific anti-OVA antibodies (total, IgG1, IgG2a, IgG2b, and IgG2c isotypes) were determined by an indirect Enzyme-Linked ImmunoSorbent Assay (ELISA), as previously described [22]. Specific anti-OVA IgE was measured with a modified ELISA, as formerly reported [19]. In all cases, a pool of sera from immunized rats was used as positive control and all data were calculated in accordance with the arbitrary units (A.U.) assigned to this pool.

Total IgA concentration from serum, gut lavages, or faecal homogenates was determined with a sandwich ELISA using a Rat IgA ELISA Quantification Set (E110-102) from Bethyl Laboratories (Montgomery, TX, USA).

### 2.6. Cytokine Quantification

MLNL (6 × 10^6^/mL) were cultured in RPMI 1640 medium supplemented with 10% heat-inactivated FBS, 100 IU/mL penicillin-streptomycin, 2 mM l-glutamine, and 0.05 mM 2-β-mercaptoethanol and stimulated with 200 mg/mL of OVA in vitro. After 72 h, supernatants were collected to assess cytokine production. 

The cytokines secreted by MLNL and from gut lavage were evaluated by ProcartaPlex^®^ Multiplex Immunoassay (Affymetrix, eBioscience, San Diego, CA, USA) according to the manufacturer’s protocol. The analysed cytokines were interleukin (IL)-10, IL-4, monocyte chemoattractant protein (MCP)-1, tumour necrosis factor (TNF)-α, and interferon (IFN)-γ, their detection limits being 11.08, 1.03, 17.99, 3.91, and 4.64 pg/mL, respectively. 

### 2.7. Statistical Analysis

Statistical analysis of the data was performed with the software package SPSS version 22.0 (IBM Statistical Package for the Social Sciences, Chicago, IL, USA). 

To assess the homogeneity of variance and the distribution of the results, Levene’s and Shapiro-Wilk tests were performed, respectively. One-way ANOVA followed by Bonferroni’s post hoc test were carried out in cases with homogenized and normally distributed variance from the data. Kruskal-Wallis and Mann-Whitney U tests were performed in cases with non-homogenized and/or non-normally distributed variance from the data. Significant differences were considered when *p* ≤ 0.05.

## 3. Results

### 3.1. Effect of Hesperidin on Food and Water Intake and Body Weight

The administration of 100 or 200 mg/kg hesperidin by oral gavage altered neither food nor water consumption in comparison to the reference group (OVAip group) (Table 1). Likewise, the inclusion of hesperidin in the food did not produce any change among groups in food or water intake (Table 2). Moreover, the administration of hesperidin, both by oral gavage or in the food, did not affect BW increase (data not shown). 

### 3.2. Effect of 100–200 mg/kg Hesperidin on Mesenteric Lymph Node Lymphocyte Composition and Functionality

**Figure 2:**
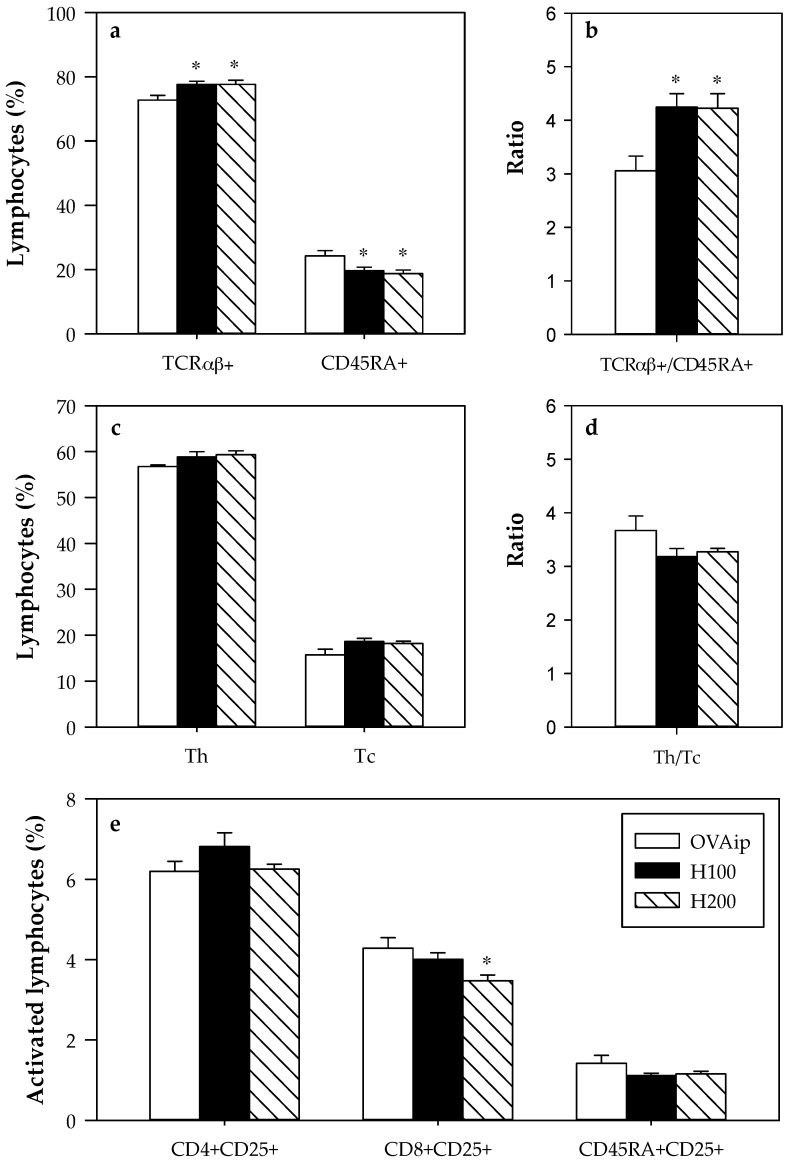
Proportion of mesenteric lymph node lymphocytes (MLNL) according to their phenotype in the first experimental design. (**a**) TCRαβ+ and CD45RA+ lymphocytes; (**b**) TCRαβ+/CD45RA+ ratio; (**c**) Th (TCRαβ+CD4+) and Tc (TCRαβ+CD8+) lymphocytes; (**d**) Th/Tc ratio; (**e**) CD25+ cells in CD4+, CD8+, and CD45RA+ lymphocytes. Data are expressed as mean ± standard error (*n* = 6). Statistical difference: * *p* < 0.05 (by Mann-Whitney U).

The influence of hesperidin administration on the lymphocyte composition of mesenteric lymph nodes was established (Figure 2). In comparison to the OVAip group, hesperidin, in both tested doses, increased the proportion of TCRαβ+ cells (107% in both doses) in MLNL and, consequently, decreased the proportion of B (CD45RA+) lymphocytes (81% and 77% for H100 and H200 doses, respectively) (Figure 2a), thus increasing the ratio of TCRαβ+/B cells (Figure 2b). The changes were not dose-dependent. No significant differences were seen in the two TCRαβ subsets, Th (TCRαβ+CD4+) and Tc (TCRαβ+CD8+) cells, meaning that both subsets were increased by hesperidin administration (Figure 2c–d). The expression of CD25 (a cell activation marker) was also determined in CD4+, CD8+, and B cells. A decrease in the proportion of CD8+CD25+ cells was observed only in the rats receiving the highest dose of hesperidin with respect to the OVAip group (Figure 2e). 

**Figure 3:**
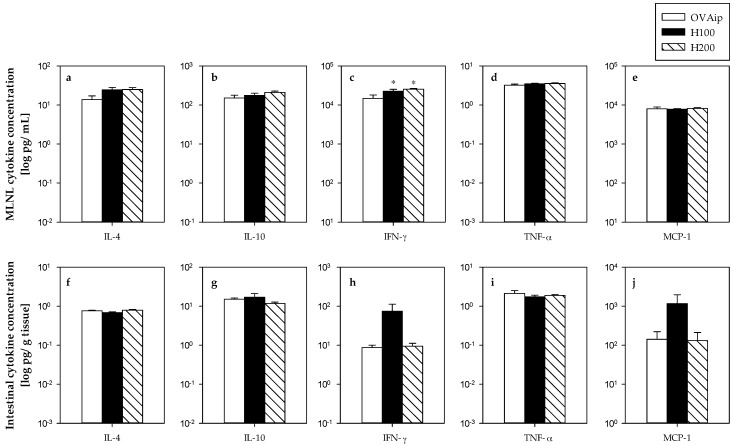
Cytokine concentrations in the second experimental design. Cytokines from stimulated MLNL (**a**–**e**) and gut lavage (**f**–**j**). Data are expressed as mean ± standard error (*n* = 6). Statistical difference: * *p* < 0.05 (by Mann-Whitney U).

To establish the function of MLNL, the cytokine pattern secreted by these cells after in vitro stimulation with OVA was determined (Figure 3a). Hesperidin administration, in both doses, induced an increase in the release of IFN-γ (145% and 150% with respect to the OVAip group, for H100 and H200 doses, respectively), a Th1-related cytokine. No differences in the secretion of IL-4, IL-10, TNF-α, and MCP-1 were observed.

In addition, cytokines in gut lavage from the first experimental design were also determined, reflecting their spontaneous secretion (Figure 3f–j). In this compartment, hesperidin did not modify the production of the considered cytokines. 

### 3.3. Effect of 100–200 mg/kg Hesperidin on Antibody Synthesis and Intestinal IgA

**Figure 4:**
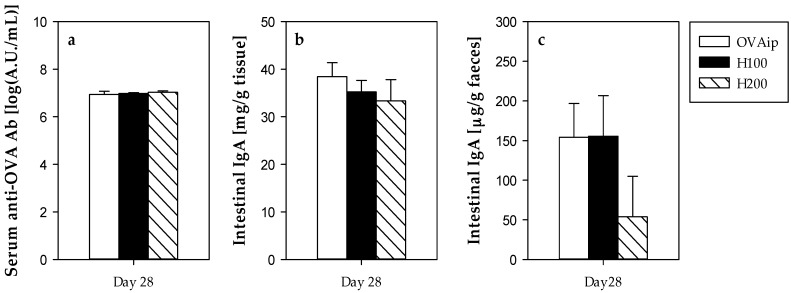
Anti-OVA antibodies (Ab) and total immunoglobulin (Ig)A levels from the first experimental design. (**a**) Serum anti-OVA Ab at the last day of the study; Total IgA from (**b**) gut lavage and (**c**) faecal homogenates from the last day of the study. Data are expressed as mean ± standard error (*n* = 6).

The untreated i.p. immunized group (OVAip) developed systemic anti-OVA antibodies (Figure 4a) and no changes were seen after hesperidin administration. No serum IgE anti-OVA antibodies were detected in any of the studied groups.

Additionally, intestinal IgA was determined in gut lavage and in faeces (Figure 4b,c) but no modifications were produced as a result of the hesperidin administration. 

### 3.4. Effect of 0.5% Hesperidin on Intestinal Lymphocyte Composition

A second experimental design was then carried out focusing on the intestinal immune system, including both inductive (MLN, PP) and effector (IEL, LPL) compartments and using an oral sensitization process that challenged these specific sites. As the hesperidin intake in the first experimental design did not affect the synthesis of anti-OVA antibodies, the second approach applied hesperidin in a more continuous manner (included in the food) and using a higher dose. Therefore, the effect of 0.5% hesperidin intake on lymphocyte composition in a rat oral sensitization model was studied, analysing the phenotype of both inductive (MLNL and PPL) and effector sites (IEL and LPL) of the GALT. 

No differences were seen as a result of the intake of the 0.5% hesperidin diet on MLNL or PPL (Table 3). In particular, in the mesenteric lymph nodes, the proportion of B (CD45RA+), T (TCRαβ+ and TCRγδ+), and natural killer (NK) cells, as well as that of TCRαβ+CD4+, TCRαβ+CD8+, TCRαβ+NK, CD4+CD25+, CD4+CD62L+, CD8+CD25+, and CD8+CD62L+ cells, was similar between the OVAoral and the H0.5 groups. Likewise, in the Peyer’s patches, the proportion of B, T, and NK cells did not differ between groups, and that of TCRαβ+CD4+, TCRαβ+CD8+, TCRαβ+NK, TLR4+ (including CD45RA+TLR4+, CD4+TLR4+, CD8+TLR4+), CD45RA+CD25+, CD4+CD25+, and CD8+CD25+ cells also remained unchanged. Interestingly, the 0.5% hesperidin diet modified the proportion of the lymphocytes in the effector sites of the GALT (Table 3). In particular, hesperidin intake increased the percentage of TCRγδ+ cells in IEL (140%) in comparison to the reference group (OVAoral group), which was due to an increase in both TCRγδ+CD8αα+ and TCRγδ+CD8αβ+ subsets, although, in this compartment, hesperidin did not significantly modify other important lymphocytes such as TCRαβ+ (and any of their CD4+, CD8+, and natural killer T –NKT- cell subsets) and NK cells. With regard to LPL, the 0.5% hesperidin diet increased the proportion of B (CD45RA+) cells to 180% and decreased that of TCRγδ+ and NK cells (35% and 29%, respectively) with respect to orally sensitized animals (OVAoral group). In addition, although the total TCRαβ+ population was not significantly modified, the percentage of TCRαβ+CD4+ cells increased (132%), whereas that of TCRαβ+CD8+ cells and NKT cells decreased (52% and 42%, respectively) with respect to the OVAoral group. Likewise, the hesperidin-enriched diet intake decreased the percentage of both CD4+ and CD8+ LPL expressing the CD103+ (50% and 60%, respectively, from that found in the OVAoral group).

### 3.5. Effect of 0.5% Hesperidin on Antibody Synthesis and Total IgA

The oral sensitization procedure applied induced the development of serum anti-OVA antibodies. However, as in the first experimental design, this immune response was not modified by hesperidin (Table 4). In order to find out what happened in Th1/Th2-associated antibody isotypes, the concentration of specific IgG1, IgG2a, IgG2b, IgG2c, and IgE antibodies was determined. The oral sensitization caused the synthesis of antibodies belonging to IgG1, IgG2a, and IgG2b isotypes, as previously reported [20,22]. The 0.5% hesperidin-enriched diet did not significantly modify the levels of these anti-OVA antibodies (Table 4). Specifically, IgG2c and IgE were not detectable in any group.

In addition, intestinal and serum IgA was assessed after four weeks of the nutritional intervention. In this approach, hesperidin intake produced an increase in intestinal IgA, whereas no changes were found with respect to serum IgA (Table 4). 

## 4. Discussion

The current study shows the effect of hesperidin in two different approaches related to Th2 immune responses to ovalbumin: an i.p. immunization with the allergen Bpt and alum, and an oral sensitization with the allergen plus cholera toxin. It was found that the administration of hesperidin in i.p. immunized rats modified MLNL composition and functionality. Moreover, in orally sensitized rats, this flavanone changed the proportions of IEL and LPL and increased intestinal IgA content. However, hesperidin did not affect anti-OVA antibody production in any of the studied immune system stimulations. 

First of all, we wanted to establish the effect of the hesperidin administration in an i.p. immunization model, triggering a systemic immune response. The hesperidin doses in this case were in accordance with the quantity given to rats to protect against gentamicin nephrotoxicity [23]. The i.p. immunization was performed using the adjuvants alum (enhancer of Th2 response [24]) and Bpt (considered a potent agent to elicit IgE response [25]), as was previously reported in Brown Norway rats [19]. However, this i.p. immunization in Lewis rats was not able to induce the production of anti-OVA IgE, contrary to when using the Brown Norway strain, a high IgE responder [26]. In addition, although a specific antibody response was induced, hesperidin did not modify the levels of such antibodies.

In order to establish the influence of hesperidin on the lymphocyte composition and function, MLNL were analysed. We observed that both doses of hesperidin used here were able to increase TCRαβ+ cell percentage and decrease that of B lymphocytes. This effect was opposite to results observed after cocoa flavonoids intake [22]. The imbalance between the proportions of TCRαβ+ cells and B cells could be due to an increase in the number of TCRαβ+ lymphocytes and/or a decrease in the number of B lymphocytes. In agreement with these results, some flavonoids have demonstrated their ability to reduce B cell viability [2,27]. In particular, catechin, a green tea flavanol, induces apoptosis of human malignant B cells [28]. Therefore, the effect of hesperidin on reducing B cell numbers may not be disregarded, although it is not reflected in antibody production. Consequently, further studies are necessary to confirm the potential of hesperidin in expanding TCRαβ+ cells or reducing B cell numbers.

Apart from the TCRαβ+ and B lymphocyte proportions, other TCRαβ+ subsets were determined. No changes in the Th (TCRαβ+CD4+) and Tc (TCRαβ+CD8+) cells were observed as a result of hesperidin administration, but a significant decrease in the proportion of CD8+CD25+ cells was found after the administration of 200 mg/kg hesperidin. The surface CD25 molecule is expressed in activated cells, and an increased number of blood T CD25+ cells in asthmatic patients has been reported [29]. Although we did not use a model of asthma, our results in terms of Tc CD25+ cell proportion would correspond with the anti-asthmatic effects of hesperidin [9]. 

On the other hand, stimulated MLNL induced the production of cytokines related to Th1 and Th2 responses. The administration of either 100 or 200 mg/kg hesperidin increased the amount of IFN-γ released from stimulated MLNL, although no changes were found in Th2 cytokines. IFN-γ is a product of Th1 cells that exerts inhibitory properties on Th2 differentiation [30], and its downregulation seems crucial for the development of allergic diseases [31]. Therefore, the increase of this type of Th1 cytokine suggests another hesperidin mechanism involved in the attenuation of allergic asthma. In addition, it has been reported that the administration of 5 mg/mL of hesperidin in a mouse model of allergic asthma inhibited the IL-4 production in splenocytes and the IL-5 concentration in the bronchoalveolar fluid [9]. Overall, these data suggest that hesperidin displays an anti-allergic action by increasing Th1 cytokines or decreasing Th2 cytokines.

Despite the results found after inducing a systemic immune response, we aimed to focus on the intestinal immune response, studying the effect of hesperidin on the GALT using a previously established model of oral sensitization [20]. As the applied doses of hesperidin did not influence antibody synthesis, we increased the dosage of flavanone, including it in the diet (0.5% hesperidin). This dosage was chosen because it has been used in previous reports, such as in the inhibition of bone loss in androgen-deficient male mice [15], in ovariectomized rats [32], and in senescent rats [33]. Considering the amount of food intake per rat, the diet with 0.5% hesperidin meant a consumption of about 360 mg/100 g BW of hesperidin per week, which was higher than that provided in the first experimental design (30 and 60 mg/100 g BW per week for 100 and 200 mg/kg hesperidin doses, respectively).

In the second approach, in contrast to the first, the hesperidin-enriched diet did not modify the composition of MLNL. Although in this second design we used a higher amount of flavanone than that used in the first approach (6- or 12-fold times higher), the inclusion of hesperidin in the food (meaning a slow intake) compared with the oral gavage (meaning a fast intake) and/or a different stimulation of MLNL (by i.p. route or by intestinal route) may affect the cellular composition of this lymphoid tissue differently. In addition, in this second approach, hesperidin did not affect the lymphocyte composition of another inductive site of the GALT, the PP. Interestingly, however, the hesperidin intake changed the proportion of cells found in the intraepithelial and the lamina propria compartments, the effector sites of the GALT. In particular, the hesperidin diet increased the proportion of TCRγδ+ lymphocytes in the intestinal epithelium, which is in line with previous results described after the intake of some polyphenol-enriched foods such as unripe apple [34] and cocoa [21]. TCRγδ+ IEL have an important role in maintaining the epithelial homeostasis, and several studies have related this cellular type with mucosa-associated tolerance [21,34,35,36,37]. However, it has been recently suggested that TCRγδ+ IEL triggered by CT exhibit antigen-presenting cell activity in recipient mice fed an oral antigen and contribute to breaking tolerance and inducing a Th2 response [38]. In particular, the oral administration of CT in mice caused the migration of TCRγδ+ IEL to LPL, where they produce IL-10 and IL-17 [38]. Our results regarding rats orally sensitized with OVA together with CT showed that hesperidin produced an opposite effect in the proportion of TCRγδ+ cells both in IEL and LPL, suggesting a protective action of hesperidin in the migration of these cells induced by CT. The hesperidin decrease in TCRγδ+ LPL is in line with the lower proportion of CD8+CD103+ cells in this compartment. The integrin CD103 (also known as αE) binds to E-cadherin and mediates T cell adhesion to the intestine [39], and is highly expressed at the mucosal sites [40], thus suggesting that CD8+CD103+ LPL would move to the intraepithelial compartment (although CD8+CD103+ IEL proportion did not significantly increase). 

In addition, after the intake of 0.5% hesperidin diet, LPL showed a relative higher proportion of B cells, which could be related to the enhanced action of hesperidin in the antibody intestinal response, thereby increasing intestinal IgA content, which is in line with other dietary polyphenols [41,42]. In addition, a rise of the Th/Tc proportion in the hesperidin-fed group was found, in agreement with the results reported for LPL after a cocoa-enriched diet using the same rat oral sensitization model [21].

Despite all these lymphocyte composition changes, the specific antibody immune response was not modified in the animals fed the hesperidin diet. In addition, when Th1- and Th2-related antibodies were studied (IgG2b, and IgG1 and IgG2a, respectively) [19], no particular effects were observed, contrary to the impact of the intake of cocoa, a rich source of flavonoids, in the same oral sensitization model [21,22]. A wide range of polyphenols has been demonstrated to play a role in decreasing specific antibody production in allergy models [8] and it has also been reported that hesperidin attenuated the OVA-specific IgE production in asthma models [9,17,43]. Nevertheless, in the current study, no differences were detected in serum anti-OVA antibodies in either the first or the second experimental designs. 

## 5. Conclusions

In summary, the present results show that hesperidin administration in i.p. immunized rats influences MLNL composition (increasing TCRαβ+ lymphocyte proportion and decreasing that of B and CD8+CD25+ cells) and functionality (increasing IFN-γ synthesis). Moreover, a diet containing 0.5% hesperidin in orally sensitized rats increases intestinal IgA content and also modifies IEL and LPL composition, suggesting the prevention of cell changes triggered by oral CT. However, this hesperidin immunomodulation is not associated with the attenuation of specific antibodies induced by both systemic and intestinal sensitization. Finally, it must be taken into account that this study shows the properties of hesperidin alone; further studies must focus on establishing the effect of hesperidin-enriched food and also the dosage of such foods to achieve these immune effects.

## Figures and Tables

**Table 1:** Food and water intake in the first experimental design. These values were established per day and per cage and referred to 100 g of the total BW in the cage. Data are expressed as the range between the two values obtained from two cages. OVAip, the reference immunized group; H100, the immunized group given 100 mg/kg hesperidin; H200, the immunized group given 200 mg/kg hesperidin.

	Food Intake (g/100 g BW/Day)			Water Intake (mL/100 g BW/Day)		
	OVAip	H100	H200	OVAip	H100	H200
Day 4	13.06–13.97	12.80–13.07	13.59–13.92	11.73–11.74	11.52–15.78	11.95–12.11
Day 11	13.51–13.57	7.75–8.64	10.20–10.47	11.31–12.66	11.26–12.39	12.15–12.74
Day 18	11.60–12.03	11.94–12.02	11.82–12.24	10.39–11.03	11.07–13.83	11.42–12.11
Day 25	9.32–9.34	9.35–9.35	9.07–9.08	11.01–11.20	12.68–14.55	13.19–14.85
Day 28	9.58–10.16	9.38–10.03	9.73–9.85	11.77–12.87	13.95–15.61	14.05–15.05

**Table 2:** Food and water intake in the second experimental design. These values were established per day and per cage and referred to 100 g of the total BW in the cage. Data are expressed as the range between the two values obtained from two cages. OVAoral, animals were fed a standard diet; H0.5, a diet containing 0.5% hesperidin.

	Food Intake (g/100 g BW/Day)		Water Intake (mL/100 g BW/Day)	
	OVAoral	H0.5	OVAoral	H0.5
Day 7	10.93–11.37	10.74–10.97	16.84–24.85	14.93–20.67
Day 14	11.65–11.70	11.28–11.52	11.39–14.45	11.05–16.23
Day 21	10.25–10.60	10.76–10.77	9.01–10.57	9.95–14.33
Day 28	8.33–8.55	7.90–8.57	9.57–10.52	8.24–12.44

**Table 3:** Proportion of MLNL, Peyer’s patches lymphocytes (PPL), intraepithelial lymphocytes (IEL), and lamina propria lymphocytes (LPL) according to their phenotype in the second experimental design. Data are expressed as mean ± standard error (*n* = 6). Statistical difference: * *p* < 0.05 (by Mann-Whitney U).

	MLNL		PPL		IEL		LPL	
Lymphocytes (%)	OVAoral	H0.5	OVAoral	H0.5	OVAoral	H0.5	OVAoral	H0.5
**CD45RA+**	12.30 ± 0.38	12.20 ± 0.29	58.90 ± 4.29	59.81 ± 3.81	10.54 ± 2.88	10.89 ± 0.88	25.86 ± 6.25	46.10 ± 6.11 *
**TCRαβ+**	77.21 ± 0.66	77.15 ± 0.72	16.16 ± 0.61	17.74 ± 1.12	41.59 ± 2.27	37.72± 3.13	29.65 ± 4.12	21.00 ± 2.78
**TCRγδ+**	1.50 ± 0.04	1.45 ± 0.03	0.67 ± 0.09	0.92 ± 0.10	11.60 ± 1.21	16.22 ± 1.63 *	3.81 ± 0.60	1.32 ± 0.40 *
**TCRγδ+CD8αα+**					73.12 ± 4.60	80.50 ± 3.39		
**TCRγδ+CD8αβ+**					26.88 ± 4.60	19.49 ± 3.39		
**NK**	0.32 ± 0.03	0.26 ± 0.03	3.31 ± 0.45	2.92 ± 0.27	24.33 ± 2.52	22.24 ± 1.82	13.81 ± 2.14	3.97 ± 1.06 *
**TCRαβ+CD4+**	77.03 ± 0.66	77.22 ± 0.62	65.28 ± 1.38	68.69 ± 1.76	22.10 ± 6.99	13.07 ± 3.60	60.45 ± 6.92	80.08 ± 5.55 *
**TCRαβ+CD8+**	22.76 ± 0.69	22.58 ± 0.49	24.73 ± 1.17	23.37 ± 1.32	71.88 ± 6.43	81.00 ± 3.45	34.63 ± 5.99	18.09 ± 1.45 *
**TCRαβ+NK**	0.61 ± 0.02	0.58 ± 0.04	6.95 ± 0.98	5.33 ± 0.59	4.95 ± 0.63	4.47 ± 0.54	3.47 ± 0.76	1.46 ± 0.23 *
**TLR4+**			37.76 ± 2.33	33.04 ± 2.74	5.43 ± 0.65	7.83 ± 1.12	6.88 ± 1.60	7.39 ± 2.41
**CD45RA+TLR4+**			41.41 ± 5.34	32.46 ± 4.10				
**CD45RA+CD25+**			3.96 ± 0.32	3.65 ± 0.61				
**CD4+CD25+**	5.58 ± 0.15	5.38 ± 0.21	10.79 ± 0.96	9.22 ± 0.86				
**CD4+CD62L+**	66.99 ± 2.15	64.42 ± 4.29			5.99 ± 3.46	1.75 ± 0.81	1.77 ± 1.09	0.81 ± 0.21
**CD4+CD103+**					91.09 ± 4.56	96.03 ± 0.98	19.02 ± 2.30	9.62 ± 1.59 *
**CD4+TLR4+**			13.41 ± 2.12	11.15 ± 1.43				
**CD8+CD25+**	3.95 ± 0.13	4.21 ± 0.32	53.01 ± 0.98	54.87 ± 3.98				
**CD8+CD62L+**	63.78 ± 2.99	60.35 ± 5.99			38.92 ± 8.70	19.18 ± 7.76	1.34 ± 0.49	1.77 ± 0.61
**CD8+CD103+**					11.83 ± 2.74	15.36 ± 2.37	77.70 ± 5.86	46.69 ± 11.97 *
**CD8+TLR4+**			36.48 ± 5.30	34.03 ± 3.89				

**Table 4:** Serum and intestinal anti-OVA and total antibodies from the second experimental design after 0.5% hesperidin diet. Data are expressed as mean ± standard error (*n* = 6). ND: not detectable. Statistical difference: * *p* < 0.05 (by ANOVA).

	Group	
	OVAoral	H0.5
Serum total Ig anti-OVA [log(U.A./mL)]	3.45 ± 0.32	3.16 ± 0.34
Serum IgG1 anti-OVA [log(U.A./mL)]	1.52 ± 0.26	1.86 ± 0.29
Serum IgG2a anti-OVA [log(U.A./mL)]	1.40 ± 0.34	1.21 ± 0.50
Serum IgG2b anti-OVA [log(U.A./mL)]	1.48 ± 0.05	1.65 ± 0.23
Serum IgG2c anti-OVA	ND	ND
Serum IgE anti-OVA	ND	ND
Serum total IgA (µg/mL)	5.72 ± 0.15	5.36 ± 0.24
Intestinal total IgA (µg/g faeces)	34.60 ± 6.79	58.07 ± 7.61 *
